# A Pilot Study to Validate a Standardized One-Week Salt Estimation Method Evaluating Salt Intake and Its Sources for Family Members in China

**DOI:** 10.3390/nu7020751

**Published:** 2015-01-22

**Authors:** Lu Zhang, Fang Zhao, Puhong Zhang, Jianmei Gao, Caixia Liu, Feng J. He, Ching-Ping Lin

**Affiliations:** 1 The George Institute for Global Health at Peking University Health Science Center, Level 18, Tower B, Horizon Tower, 6, Zhichun Road, Haidian District, 100088 Beijing, China; E-Mails: zhang.lu@imicams.ac.cn (L.Z.); fzhao@georgeinstitute.org.cn (F.Z.); clin1@georgeinstitute.org.cn (C.-P.L); 2 Center for Health Policy and Manag ement/Institute of Medical Information, Chinese Academy of Medical Sciences, 3, Yabao Road, Chaoyang District, 100020 Beijing, China; 3 Huairou Center for Disease Control and Prevention, 23, Fule North Street, Huairou District, 101400 Beijing, China; E-Mails: hrgaojm@sina.com (J.G.); hrcdcmb@yeah.net (C.L.); 4 Wolfson Institute of Preventive Medicine, Barts and The London School of Medicine and Dentistry, Queen Mary University of London, Charterhouse Square, EC1M-6BQ London, UK; E-Mail: f.he@qmul.ac.uk

**Keywords:** salt intake, methods, urine collection, accuracy assessment

## Abstract

The objective of this study was to develop a new method named the “one-week salt estimation method” that could estimate an individual’s salt intake and the sources of salt in the diet, and to evaluate this new method with a 24-h urine collection. The new method estimates salt intake from: (1) household cooking by weighing the family salt container and other high-salt condiments or sauces at the beginning and end of a week; (2) processed food according to established China food composition figures; and (3) cafeteria or restaurant meals using the results of previous studies. Consumption of salt additives and major salt contained foods and salt intake related eating habits were collected using a structured simple seven-day questionnaire. In order to validate the method, we studied 37 individuals from 11 families using the new method and 26 of these participants collected seven concurrent 24-h urine samples. The average salt intake for the 26 participants was 15.6 ± 5.5 g/person/day (mean ± standard deviation) by the 24-h urine collection and 13.7 ± 6.5 g/person/day by the new method. The difference was 1.8 ± 4.2 g/day/person (*p =* 0.037). The Pearson correlation coefficient was 0.762 (*p <* 0.001) and the partial correlation coefficient was 0.771 (*p <* 0.001) when adjusted for family code. Bland-Altman Plot showed the average of the difference between the two methods was −1.83, with 95% limits of −10.1 to 6.5 g/person/day. The new method showed that 43.7% of salt intake came from household cooking (33.5% from cooking salt, 10.2% from other condiments and sauces), 12.9% from processed food, and 43.4% from eating out. In conclusion, despite its limitations of underestimating salt intake, the “one-week salt estimation method” is easier for people to implement and is likely to provide useful information that highlights the excessively high intake of salt and its sources, and in turn is helpful in guiding dietary salt reduction.

## 1. Introduction

A high salt intake is related to hypertension and cardiac-cerebral vascular disease, the leading cause of death in China [1]. The WHO recommends the consumption of less than 5 g salt (or 2 g sodium) per person per day [2]. The Chinese government has been making great efforts in salt intake surveillance [3] and salt reduction through healthy lifestyle campaigns [4]. However, salt intake in China remains very high. Indeed, it is one of the highest in the world with an average intake of approximately 12 g/day/person [5,6,7]. Although salt intake surveillance data are available, people seldom know the exact amount and sources of salt intake in their own diets. The WHO recommends a process for dietary salt reduction: (1) estimate salt intake; (2) identify the major sources of salt intake; and (3) propose measures to reduce the intake [2]. Our study has developed a method that could estimate the amount of salt consumption and sources of salt in the diet for individuals to help them take appropriate measures to reduce salt intake. This manuscript addresses the development and validation of the salt intake estimation method.

Several methods can be used to estimate salt intake: timed or spot urine sodium measurements, duplicate diets and dietary surveys [2]. 24-h urine collection is regarded as the gold standard for estimating salt or sodium intake; however, it creates a high participant burden and also has challenges with completeness of the specimen [8,9,10,11,12,13,14]. Overnight urine collection is well-correlated with 24-h urinary collection: the correlation coefficient has been reported to be 0.710 [8,9,10] and is recommended as a good alternative [8,9,13,15], but it is less useful for estimating absolute levels of daily salt intake due to diurnal variation of sodium excretion. The duplicate diet method involves duplicating and saving a portion of all food and drink consumed throughout a day, which is then sent for a complex laboratory analysis to identify its nutrients. It is a challenge to duplicate diets consumed during the course of a whole day by participants. Finally, dietary surveys, such as 24-h dietary recall, can measure salt intake without laboratory analysis using a food composition table combined with a record of salt added during cooking and at the table [10]. However, INTERMAP reported a correlation of only 0.42 between 24-h urine collection and 24-h dietary recall and this correlation coefficient was 0.33 for China–even poorer than for other countries [16]. So, dietary survey alone has been proven to be unreliable, with poor correlation with urine collection. [7,10].

Due to their methodological complexity and inaccuracy, traditional urine collection, duplicate diets and dietary surveys are therefore not useful for evaluating salt intake for individuals. A reliable non-laboratory technique is needed to estimate salt intake and sources of salt in the diet, and find targeted measures for individuals to reduce their salt consumption. In China, salt intake mainly comes from cooking rather than processed food [2,7]. Therefore China is a good setting for developing a simple and standardized method for measuring salt intake based on weighing salt and major salt containing foods. This study was conducted in an attempt to develop a new method to help professionals and non-professionals to estimate the amount and analyze the sources of salt intake at individual level in China where salt added to food during cooking is the major source of salt intake [2,7].

## 2. Methods

### 2.1. Principles of the One-Week Salt Estimation Method

The “one-week salt estimation method” is a way of estimating individuals’ salt intake and identifying the sources of salt in the diet. This new method estimates salt intake from three sources: household cooking, processed food, and cafeterias or restaurants.

**(a). Household cooking.** We recorded salt and other sodium-containing condiments (such as monosodium glutamate, soy sauce, vinegar and other sauces) used in household cooking by weighing them at the start and end of the week [17]. This provided the total salt consumption for the whole household, which was then assigned to individuals. All the salt and sodium-containing condiments were converted into salt intake according to China Food Composition data [18].

**(b). Processed food consumed at home.** We estimated sodium intake from processed food and converted it into salt (sodium chloride) intake. We classified processed food into six categories: (1) instant noodles; (2) deep-fried dough sticks and cake; (3) steam stuffed bun, dumplings, baked pancake, bread; (4) processed meat; (5) salted vegetables; and (6) others. The amounts of food consumed for each category were recorded by participants at the time of consumption. An arithmetic average sodium content for each of the six categories of processed food was used to calculate the salt intake and combining with the recorded consumption according to China Food Composition data [18]. 

**Allocation of household salt intake to individuals.** On average an individual’s salt intake is consistently proportional to his/her energy intake or the total amount of food consumed, *i.e.*, the higher the total food consumption, the higher the salt intake. Bu et al showed that energy intake has a linear relationship with sodium intake (*r =* 0.702) in a Korean population eating an Asian diet [19]. We calculated the energy intake of each family member from their age, body weight and gender using the Recommended Nutrient Intake (RNIs) of energy in the China dietary nutrition survey reference intake scale [20], and then used the calculated energy intake to allocate the total household salt intake to each household member.

**(c). Eating Out.** We estimated the salt consumed from eating out (at cafeterias or restaurants) using a previous study which showed that in China, salt consumed from eating out was about 1.5 times the salt consumed through household cooking [21,22]. The SMASH baseline survey was a population-based cross-sectional survey in Shandong province, China, which showed that total average daily dietary sodium intake was 5745 mg estimated by the combination of weighing condiments in the household and 24-h dietary recall [21]. This survey also investigated consumption of condiments at restaurants. The average consumption of salt, soy sauce, flour sauce and monosodium glutamate was 6.4 g/person/meal, 7.1 g/person/meal, 2.4 g/person/meal, and 2.0 g/person/meal, respectively [22]. We used the sodium reference values from China Food Composition Table 2002 [18] to calculate the sodium content for all condiments, the average consumption of sodium was 3139 mg per meal. The SMASH study provides data for a single meal, which we used to estimate sodium intake for those eating out for three meals a day. Assuming the sodium consumption was equal for three meals, the average daily consumption of sodium for a person eating at restaurant was estimated to be 9417 mg. So the ratio of sodium intake at restaurant *vs.* at home was 1.6 (9417/5745). The Chinese Nutrition Society estimates the proportion of nutrient intake from breakfast, lunch and dinner is about 3:4:3 [23]. Therefore, the average daily consumption of sodium for a person eating all three meals at a restaurant was estimated to be 7848 (3139/0.4) mg to 10,463 (3139/0.3) mg. The ratio of sodium consumption in restaurants to sodium consumption at home can therefore be calculated as 1.4 to 1.8. In the current study, we used a relatively conservative number of 1.5.

**Figure 1:**
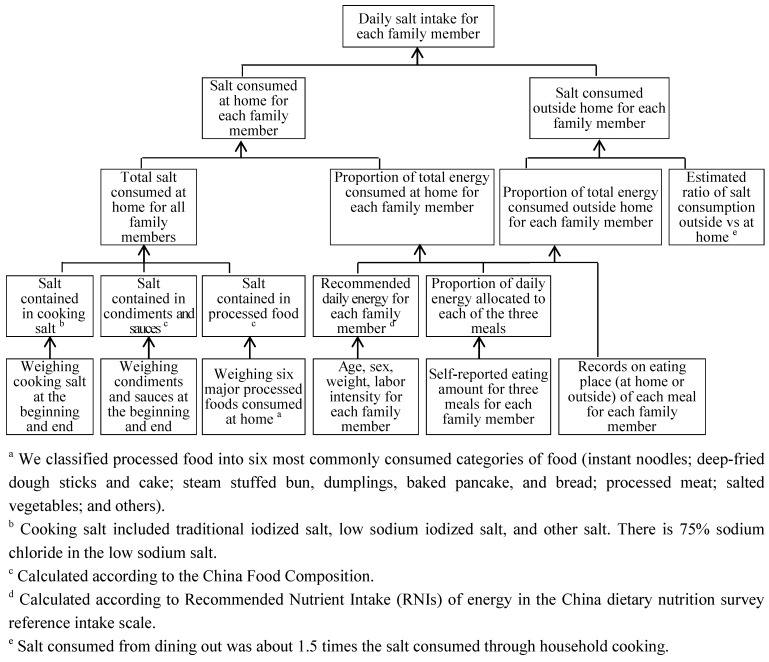
The pathway of the one-week salt estimation method in measuring individual salt intake.

The pathway of the new method was shown in Figure 1.

We selected a period of one week to cover both weekday and weekend, so that the salt intake results could be more representative of an individual’s usual intake. We refer to our method as “household-based” because it estimates the salt consumption of an entire household by collecting dietary record for all family members (those who live and eat within the household) during the investigation period.

Unlike the traditional dietary record method, we used a simple questionnaire (called the One Week Salt Intake Recording Form) to collect the information needed for the new method which mainly focused on major sodium-containing food and eating habits that influence salt intake. The information collected included all family members’ age, body weight, sex, labor intensity, weight of cooking salt/condiments and sauces at the start and end of the week, weight of major processed food (recorded and weighed by participants when consumed), discarded food ratio of the family (reported by the main cook) reflecting the proportion of salt used during cooking but discarded when eating, and detailed records of eating at home or not for the entire household as well as guests for each meal each day during the study week. We devised an online software program to support data collection, automated calculations, results reporting, alerts and suggestions.

### 2.2. Comparison between the “One-Week Salt Estimation Method” and 24-h Urine Collection

To validate the method, we compared the new method with the 24-h urine collection which is considered as “gold standard method”. Parallel 24-h urine collection was made using a standardized protocol (see “Data collection” part below for detail) to estimate salt intake during the one week period. The study was carried out in Huairou District, Beijing. Guidelines for the ethical Declaration of Helsinki were followed and all procedures involving human subjects were approved by Biomedical Ethics Committee of Peking University in 2012. Each participant was given appropriate explanation before signing the consent form. The children and their guardian signed the consent form.

### 2.3. Participants

We recruited participating households through a primary school as the 7-day 24-h urine collection for children would need supervision by the teachers when children were attending school. Our inclusion criteria was: (1) Willing and able to collect 24-h urine for 7 consecutive days; (2) For women, not be in their menstrual period during the urine collection; and (3) Willing not to travel too far during the collection period. Individuals who had diabetes or nephropathy, were on diuretics or bedridden were excluded. To minimize disruption of the school, we selected 11 households from one class. 37 members from the 11 households agreed to participate, and 26 of them met the inclusion criteria for the seven days’ 24-h urine collection and completed the urine collection.

### 2.4. Data Collection

Data collection was carried out by well-trained investigators of local CDC (Huairou Center for Disease Control and Prevention) in April 2012. Before the study began, we convened all family members in the school for training and provided each family with a SF-400 kitchen scale with accuracy of 1 gram and a set of urine collecting instrument and a backpack.

During the consecutive 7-day investigation, a designated family member weighed the food and completed the One Week Salt Intake Recording Form and the investigators measured 24-h urine volume and collected urine samples. Investigators visited each family at the same time each evening to check the completeness of urine and dietary record and gave participants specific instructions to avoid mistakes or bias. To ensure the completeness of 24-h urine collection, investigators repeated 24-h urine collection instructions during each visit, and sent short text message reminders to the adult participants and teachers’ mobile phones. The school allocated special places for the children to collect urine. Investigators collected urine aliquots every evening and refrigerated them at −20 °C. They recorded detailed information such as vomiting, diarrhea and sweating during urine collection for all participants. During the study, two adults withdrew from the urine collection for personal reasons and 26 completed the urine collection.

For the urine analysis, sodium was measured by ion selective electrode using an AC9000 electrolyte analyzer and creatinine measured by the Jaffe method using a Hitachi 7080 automatic biochemistry analyzer. Quality control was conducted by comparing results of 5 anonymous duplicates that showed a 0.995 Intra-class Correlation Coefficient. The completeness of the urine was assessed by urine creatinine. The amount of salt (g) was calculated by multiplying sodium with 2.54.

### 2.5. Data Analysis

We used SPSS 18.0 for data analysis. Continuous variables were presented as means and standard deviation and categorical variables were presented as proportions. We used Bland-Altman plot to illustrate the agreement between the two methods, Pearson correlation as well as partial correlation when adjusted for family code as a nominal variable to describe correlation, and t-test to examine the difference between the two methods.

## 3. Results

### 3.1. Basic Characteristics

Of the 37 participants from 11 households, all completed the dietary record, 28 met the inclusion criteria for urine collection and 26 finished the 7 days 24-h urine collection successfully. Table 1 shows the characteristics of the participants.

**Table 1:** Characteristics of child and adult participants.

Characteristics	Children	Adults
Age range	2–11	34–74
Participants who completed the dietary record (*n =* 37)		
Number	12	25
Male (%)	6 (50.0)	11 (44.0)
Age (mean ± SD)	10.0 ± 3.2	42.3 ± 9.4
Weight (Kg) (mean ± SD)	36.7 ± 11.3	69.0 ± 14.1
Participants who completed the 7-day 24-h urine collection (*n =* 26)		
Number (%)	10	16
Male (%)	5 (50.0)	8 (50.0)
Age (mean ± SD)	10.1 ± 0.3	39.4 ± 3.0
Weight (Kg) (mean ± SD)	37.1 ± 5.8	70.1 ± 11.8

### 3.2. 24-h Urine Collection

The average 24-h urine volume, creatinine, sodium and potassium excretion during the one week investigation for the adults and children were presented in Table 2.

**Table 2:** Urine volume and excretion of creatinine, sodium and potassium by consecutive 7-day 24-h urine collection.

Age Group	*N*	Urine Volume	Creatinine Excretion	Sodium Excretion	Potassium Excretion	Sodium-Potassium Ratio
		(mL/24 h)	(mmol/24 h)	(mmol/24 h)	(mmol/24 h)	(mmol:mmol)
Total	26	1678.1 ± 678.5	10.8 ± 4.5	268.5 ± 94.4	55.8 ± 23.3	5.2 ± 1.5
Adults	10	2078.7 ± 529.0	13.8 ± 2.9	330.4 ± 54.6	68.9 ± 18.9	5.1 ± 1.3
Children	16	1037.1 ± 265.7	6.0 ± 1.0	169.4 ± 45.3	34.8 ± 10.6	5.2 ± 1.8

### 3.3. Comparison between One-Week Salt Estimation Method and 24-h Urine Collection

**Figure 2:**
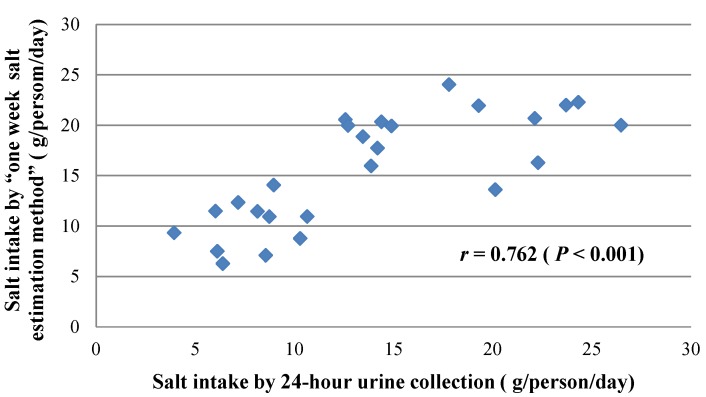
Person correlations between salt measured by 24-h urine collection and “one-week salt estimation method” (*n =* 26).

The mean salt intake calculated from 24 h urinary sodium was 15.6 ± 5.5 g/day/person and it was 13.7 ± 6.5 g/day/person by the new method. The correlation coefficient between the two methods was 0.762 (*p <* 0.001, Figure 2) and the partial correlation coefficient was 0.771 (*p <* 0.001) by controlling the family code as a nominal variable. Salt intake measured by 24-h urine method was 1.8 ± 4.2 g/day/person higher than that by the new method (*p =* 0.037). The difference for adults and children was 1.7 ± 5.0 and 2.0 ± 2.7 g/day/person respectively (Table 3).

Because of the possibility of incompleteness of the urine collection, we recalculated the correlation with the assumption that daily urine creatinine excretion was relatively stable during the 7 days. For those participants whose daily creatinine excretion was lower than the7-day average by 30%, the urine collection was considered incomplete or suspicious. For these participants, we used two methods to recalculate the correlations: (1) exclude the possible incomplete 24-h urine collections; or (2) replace the data with the average. There were three participants (2 adults and 1 child), each for only one day, that had a daily creatinine excretion lower than their own 7-day averages. The adjusted and differences were 0.805 (*p <* 0.001) and 1.9 ± 4.0 g/24 h excluding their data, and 0.756 (*p <* 0.001) and 2.0 ± 4.3 g/24 h replacing their daily data with their average.

**Table 3:** Difference of salt intake measured by 24-h urine collection and “one-week salt estimation method” (*n =* 26).

Method	Estimated Salt Intake (g/Day, Mean ± SD)		
	Children	Adults	Total
7 days 24-h urine collection (standard method)	9.8 ± 2.4	19.2 ± 3.2	15.6 ± 5.5
One-week salt estimation (new method)	7.8 ± 2.1	17.5 ± 5.4	13.7. ± 6.5
Difference	2.0 ± 2.7	1.7 ± 5.0	1.8 ± 4.2
	*p =* 0.044	*p =* 0.193	*p =* 0.037

**Figure 3:**
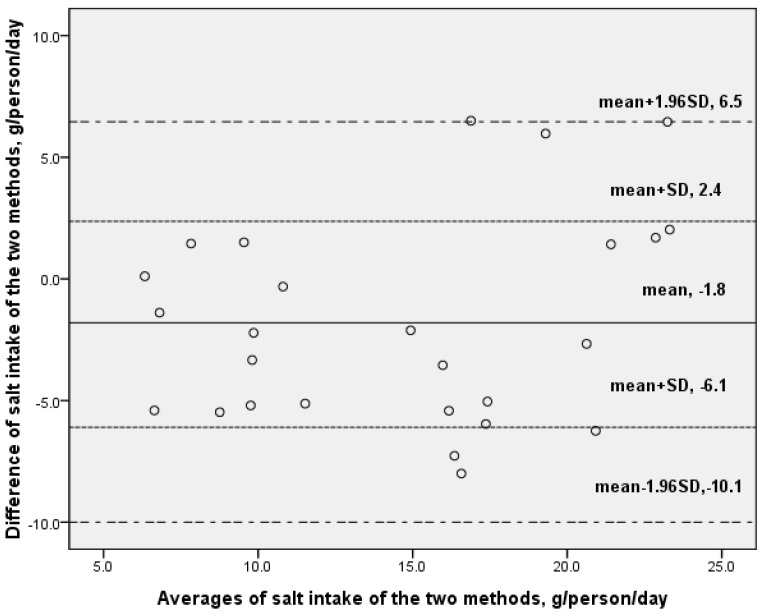
Difference against averages of salt intake estimated by 24-h urine collection and one-week salt estimation methods (Bland-Altman analysis; *n* = 26).

According to the Bland-Altman plot (Figure 3), the mean difference between salt intake estimated by 24-h urine collection and the new method was −1.83, the limits of agreement of 95% (mean ± 1.96 SD) show a large variation, from −10.1 to 6.5. No correlation was found between the average and difference of the two methods.

### 3.4. Results of the “One-Week Salt Estimation Method”

Table 4 presents the salt intake and the sources of salt in the 37 participants. The daily salt intake was 13.6 ± 6.7 g/person/day for all participants, and 7.6 ± 2.9 g/person/day for children, 16.5 ± 6.1 g/person/day for adults. Taking all participants together, 43.7% of salt intake came from household cooking (33.5% from cooking salt, 10.2% from other salt containing condiments and sauces), 12.9% from processed food, and 43.4% from eating out. In other words, 87.1% of the salt came from cooking (household cooking and cafeteria or restaurant cooking) and only 12.9% came from processed food.

**Table 4:** Daily salt intake and its sources—One-week salt estimation method (*n =* 37).

Family Members	Daily Salt Intake (g/person, Mean ± SD)	Proportion of the Different Sources (%)			
		Cooking Salt Added	Condiments and Sauces	Restaurants or Canteen	Processed Food
Total (*n =* 37)	13.6 ± 6.7	33.5	10.2	43.4	12.9
Male (*n =* 17)	15.7 ± 7.1	28.4	9.1	51.2	11.3
Female (*n =* 20)	11.9 ± 6.0	37.9	11.2	36.7	14.2
Children (*n =* 12)	7.6 ± 2.9	35.1	11.0	40.3	13.5
Boy (*n =* 6)	8.6 ± 2.4	30.7	10.3	44.8	14.2
Girl (*n =* 6)	6.6 ± 3.1	39.6	11.7	35.9	12.9
Adults (*n =* 25)	16.5 ± 6.1	32.8	9.8	44.8	12.6
Male (*n =* 11)	19.6 ± 5.6	27.2	8.4	54.7	9.7
Female (*n =* 14)	14.1 ± 5.5	37.2	11.0	37.1	14.8

## 4. Discussion

Accurate assessment of dietary salt intake has long been a challenge. 24-h urine collection is considered as the “gold standard”, but has obvious limitations such as high participant burden and over- or under-collection. When compared to the traditional dietary record, the new method which we have developed has lower subject burden, can be conducted by non-professional persons, and is more accurate in estimating salt intake. The results of this study showed that the “one-week salt estimation method” had a correlation of 0.762 with 24-h urine collection, which is higher than the previously reported 0.42 for 24-h diet recall [7] and similar to the correlation of 0.710 between 24-h and overnight urine collection [10].

In our study, we recruited the participants with family as a unit. The whole family including both children and adults were enrolled to ensure that the range of the salt intake was wide enough to show the correlation between the gold standard and the new method. Due to the wide-range of salt (NaCl) and sodium (Na+) sources such as sodium present in natural food or sodium added through processed food or cooking [2], the correlation coefficient for Na was consistently lower than the single nutrients such as protein [7]. This reflects the special difficulties in accurately assessing salt intake using dietary survey methods [7]. The new method requires no laboratory analysis, and the information needed is simple and understandable to non-professionals. Although the calculation is complex, the data collection required by the new method is very simple. With the aid of computational software, this method can be easily used to calculate the amount and sources of salt for each family member without guidance from trained staff. Besides the web-based software, a smart phone application could also be developed to support its utility not only in salt intake estimation in larger studies but also for individual families or individuals to remind and support them to reduce salt intake.

The “one-week salt estimation method” is useful in identifying the sources of salt in the diet and has the potential to estimate the amount of salt consumed at a household or individual level. The salt intake of the 26 people using 24-h urine collection was 15.6 g/person/day compared to 13.7 g/person/day for the 37 people using the new method. Both of these results are higher than 12 g/person/day, the result of Chinese resident nutrition and health survey in 2002 [5]. The higher salt intake may be related to the fact that all the participants came from a rural district of Beijing where more salt is consumed than in urban areas [7,24]. However, the possibility of underestimation by 2002 national survey also exists, and further validation work for salt intake estimation should be done in the future. According to the salt source analysis, salt intake mainly comes from cooking suggesting that in countries like China, education and policy in salt reduction should focus on cooking at home as well as cafeterias or restaurants. Specific recommendations must be made according to the detailed results of the salt sources.

Our study showed that the new method under-estimated salt intake by 1.8 g/person/day compared with 24-h urine collection. This underestimation may be due to two reasons. First, 10%–12% of sodium intake derived from Na is inherent to natural food [25] so the new method, by only estimating salt intake from processing or cooking, did not take into consideration the sodium inherent in natural food. This part of underestimation was predictable and quantifiable because it was homogeneous in our study between the two methods. So when evaluating the absolute level of an individual’s salt intake, it would be more appropriate to take into account the underestimation by the new method. Second, in this study, the simple dietary questionnaire only included the most common types of processed food consumed by the Chinese, such as instant noodles, dumplings, deep-fried dough sticks, salted vegetables and salted meat. The difference in sodium intake could be from consumption of other processed foods that were not included in our questionnaire. Additionally, there may be a difference between the salt content of the processed food and the average salt level estimated from the Food Composition Table for the six categories. This might cause further discrepancy in salt intake estimated from the two different methods.

The new “one-week salt estimation method” has its limitations. Bland-Altman analysis is a method to examine the agreement between the new method and the standard method, and also to test the variability in those differences across individuals. If the two methods are in agreement, these differences will be homogeneous and the points will be dispersed along the average line. The widespread of the points and large variation of the 95% limits of agreement is a sign of disagreement [26]. Bland-Altman Plot showed a large variation of the difference between the new method and the 24-h urine collection which is the standard method in salt intake measurement. Unlike the duplicate diets method, the new method can only be used to estimate additives such as salt and oil (the estimation of oil intake is similar to salt) while it cannot produce a map of other nutrient intake information. The correlation between the new method and the gold standard 24-h urine collection largely depends on the proportion of salt intake from household cooking salt. The higher the proportion of cooking salt, the higher the correlation, and the more reliable this method is for salt intake estimation because the estimation of salt intake during eating out is roughly based on an overall estimated ratio of salt intake between eating out and eating at home. As a result, the validity of this method may largely depend on dietary habits and cultural factors. Further evaluation should be done before it can be used in countries where salt intake comes mainly from processed foods and eating out. In our study, due to the limited sample size, the estimation of salt intake and sources was shown as an example rather than describing the exact map of the whole population. In addition to the underestimation of the new method by 1.8 g/d compared to the 24-h urine collection, further underestimation might exist because the 24-h urine collection method itself has underestimated salt intake by approximately 10% due to losses via the skin and gastric system. For the new method, further underestimation may be due to the under reporting of salt-containing sources or processed foods. Methodologically, further study with larger sample size should be conducted to establish salt source apportionment model based on individual’s demographic dietary information, which might be better than the current semi-quantitative approach.

Despite the limitations of this study including the small sample size, it confirms that the salt intake of the study population is twice the recommended level. Almost everyone in China should be reducing salt intake. However, most people in China do not know the exact amount and sources of their salt intake. In addition to traditional health education on the harm of high salt intake, easy self-administered salt intake measurement tools should be developed to support individuals in salt intake evaluation and to help find out the most effective ways to reduce salt intake.

## 5. Conclusions

Despite an underestimation of salt intake, this carefully conducted pilot study showed that the “one-week salt estimation method” was useful in identifying the sources of salt in the diet and had a lower subject burden when compared to 24 h urine collection and traditional dietary record method. Larger study needs to be done to improve and validate its accuracy in various populations.
